# Disrupting Reconsolidation by Systemic Inhibition of mTOR Kinase via Rapamycin Reduces Cocaine-Seeking Behavior

**DOI:** 10.3389/fphar.2021.652865

**Published:** 2021-04-09

**Authors:** Fushen Zhang, Shihao Huang, Haiyan Bu, Yu Zhou, Lixiang Chen, Ziliu Kang, Liangpei Chen, He Yan, Chang Yang, Jie Yan, Xiaohong Jian, Yixiao Luo

**Affiliations:** ^1^ Key Laboratory of Molecular Epidemiology of Hunan Province, School of Medicine, Hunan Normal University, Changsha, China; ^2^ Yiyang Medical College, Yiyang, China; ^3^ Department of Forensic Science, School of Basic Medical Science, Central South University, Changsha, China

**Keywords:** mTOR, rapamycin, reconsolidation, drug memory, self-administration

## Abstract

Drug addiction is considered maladaptive learning, and drug-related memories aroused by the presence of drug related stimuli (drug context or drug-associated cues) promote recurring craving and reinstatement of drug seeking. The mammalian target of rapamycin signaling pathway is involved in reconsolidation of drug memories in conditioned place preference and alcohol self-administration (SA) paradigms. Here, we explored the effect of mTOR inhibition on reconsolidation of addiction memory using cocaine self-administration paradigm. Rats received intravenous cocaine self-administration training for 10 consecutive days, during which a light/tone conditioned stimulus was paired with each cocaine infusion. After acquisition of the stable cocaine self-administration behaviors, rats were subjected to nosepoke extinction (11 days) to extinguish their behaviors, and then received a 15 min retrieval trial with or without the cocaine-paired tone/light cue delivery or without. Immediately or 6 h after the retrieval trial, rapamycin (10 mg/kg) was administered intraperitoneally. Finally, cue-induced reinstatement, cocaine-priming-induced reinstatement and spontaneous recovery of cocaine-seeking behaviors were assessed in rapamycin previously treated animals, respectively. We found that rapamycin treatment immediately after a retrieval trial decreased subsequent reinstatement of cocaine seeking induced by cues or cocaine itself, and these effects lasted at least for 28 days. In contrast, delayed intraperitoneal injection of rapamycin 6 h after retrieval or rapamycin injection without retrieval had no effects on cocaine-seeking behaviors. These findings indicated that mTOR inhibition within the reconsolidation time-window impairs the reconsolidation of cocaine associated memory, reduces cocaine-seeking behavior and prevents relapse, and these effects are retrieval-dependent and temporal-specific.

## Introduction

Drug addiction is defined as a chronic psychiatric disease with a high rate of relapse even after a long period of withdrawal. Addicted individuals experience intense drug craving that can persist long after withdrawal, leading to high relapse rates. Additionally, they exert compulsive seeking and drug-taking behaviors that are difficult to control despite the serious adverse consequences of drug abuse. The critical problem of drug addiction is to prevent and reduce relapse after withdrawal (Grant et al., 1996; Childress et al., 1999; Kilts et al., 2001). To date, there are no available pharmacological or non-pharmacological therapies to treat drug addiction and prevent relapse completely (Nestler, 2001; Hyman et al., 2006; Volkow and Boyle, 2018; Ahmed et al., 2020). Much evidence shows that the persistence of drug memory and the difficulty in eliminating are the root causes of compulsive drug use behavior, drug-seeking behavior, and relapse after withdrawal (Childress et al., 1986; Kelley, 2004; Torregrossa et al., 2011). Hence, elucidating the neurobiological mechanisms of drug memory and disrupting it through various interventions seem to be an effective way to treat drug addiction and prevent relapse after withdrawal.

The formation of conditioned associations between repeated drug abuse and associative context is essential for the generation and maintenance of addiction-related behaviors. Exploring the neural mechanisms underlying the associated learning process in the formation of drug memories will help us understand addictive behaviors from the perspective of maladaptive learning and memory and develop new treatments to prevent relapse (O’Brien et al., 1998). Chronic use of addictive drugs leads to adaptive changes in plasticity in the central nervous system, which may be related to the pathological memories formed between repeated exposure to drugs and drug-associated cues (Everitt et al., 1999; Jentsch and Taylor, 1999). There are similar processes between drug memory and normal learning memory, such as consolidation and reconsolidation (Dudai, 1996; McGaugh, 2000; Nader et al., 2000; Sara, 2000; Nader and Hardt, 2009).

Consolidation and reconsolidation are specific phases in which the memory becomes unstable after acquisition or reactivation and can be disrupted, requiring *de novo* protein synthesis to be restabilized (McGaugh, 2000; Sara, 2000; Nader and Hardt, 2009). Interfering with consolidation or reconsolidation can disrupt drug memory and consequently attenuate the drug seeking or reinstatement in gold-standard animal models of addiction (Hellemans et al., 2006; Robbins et al., 2008). Drug memory can be activated during the reconsolidation process by controlled re-exposure to drug-context or cues, which provides operability to interfering with drug memory. Current research on drug memory is mainly focused on the reconsolidation (Lee et al., 2005; Milton et al., 2008a; Milton et al., 2008b; Taylor et al., 2009; Sanchez et al., 2010; Wells et al., 2013). Interfering with reconsolidation is a promising strategy for the treatment of drug addiction and relapse prevention.

The mammalian target of rapamycin (mTOR) signaling pathway is well known to exert complex physiological functions *in vivo*, especially, plays a critical role in gene transcription regulation and protein translation initiation via modulating downstream target proteins (Sabatini et al., 1994; Schmelzle and Hall, 2000; Laplante and Sabatini, 2012). Reconsolidation is a protein synthesis-dependent process (Nader et al., 2000; Dudai, 2006). Therefore, the activity of mTOR probably regulates reconsolidation of drug memories. Previous studies have shown that inhibition of the activity of mTOR signaling pathway can disrupt spatial memory consolidation, as well as the consolidation and reconsolidation of contextual fear memory (Dash et al., 2006; Parsons et al., 2006). The mTOR signaling pathway has also been reported to be involved in reconsolidation of drug memories in conditioned place preference (CPP) and alcohol self-administration (SA) animal models (Barak et al., 2013; Lin et al., 2014). However, whether or not disruption of reconsolidation by mTOR inhibition could inhibit drug-seeking behaviors and prevent relapse still remains largely unknown.

It is generally accepted that rapamycin is the specific inhibitor of mammalian target of rapamycin (Brown et al., 1994; Sabatini et al., 1994; Abraham and Wiederrecht, 1996). In the present study, we investigated the role of mTOR via rapamycin in the reconsolidation of cocaine-associated memory using a cocaine self-administration model. We also assessed the effects of mTOR inhibition on cue-induced- and cocaine priming-induced reinstatement of drug seeking and on spontaneous recovery of cocaine-seeking.

## Materials and Methods

### Subjects

Male Sprague Dawley rats (weighing 260–280 g on arrival) were obtained from the Tianqin Laboratory Animal Technology Co. Ltd., China. Rats were housed in groups of five under controlled temperature (23 ± 2°C) and humidity (50 ± 5%), and maintained on a 12 h light/dark cycle (lights off at 8:00, lights on at 20:00) with access to food and water ad libitum. All of the rats were handled 3 min per day for 5 days before the surgeries. All animal procedures were performed in accordance with the Guide of Hunan province for the Care and Use of Laboratory Animals, and the experiments were approved by the Local Committee on Animal Care and Use and Protection of the Hunan Normal University. All the experiments were performed during the dark phase.

### Intravenous Surgery

Rats (weighing 300–320 g at the time of surgery) were anesthetized with sodium pentobarbital (60 mg/kg, i. p.). Catheters were inserted into the right jugular vein with the tip terminating at the opening of the right atrium as described previously (Lu et al., 2005). The cannulae were anchored to the skull with stainless steel screws and dental cement. A stainless steel stylet blocker was inserted into each cannula to maintain patency and prevent infection (Xue et al., 2012). Rats were then housed individually for 5–7 days to recover before the training sessions began.

### Behavioral Procedures

#### Intravenous Cocaine Self-Administration Training

The operant chambers used (AniLab Software and Instruments, Ningbo, China) were equipped with two nosepoke operandi (AniLab Software and Instruments, Ningbo, China) located 5 cm above the chamber floor. Nosepokes in the left side (active) operandum led to cocaine infusions that were accompanied by a 5 s tone-light cue. Nosepokes in the right side (inactive) operandum were also recorded but had no programmed consequences. Rats were trained to self-administer cocaine hydrochloride (0.75 mg/kg/infusion) during three 1 h daily sessions separated by 5 min over 10 days. The sessions began at the onset of the dark cycle (8:00–20:00). A fixed-ratio one reinforcement schedule was used, with a 40 s timeout period after each infusion. Each session began with the illumination of a houselight that remained on for the entire session. The number of drug infusions was limited to 20 per hour to prevent death by overdose (Xue et al., 2012; Luo et al., 2015). We excluded a total of nine rats from the experiments: four rats due to catheter patency failure and five rats due to failure to acquire cocaine self-administration.

#### Nosepoke Extinction

During extinction, the conditions were the same as during cocaine self-administration training, except that cocaine was no longer available and without the tone-light cue (Shi et al., 2015).

#### Retrieval Trial

A 15 min retrieval to reactivate cocaine-associated memories began 24 h after the last nosepoke extinction session. The retrieval conditions were the same as during cocaine self-administration training except that active nosepokes with no reward cocaine.

#### Cue Extinction

During extinction, the conditions were the same as during cocaine self-administration training, with the exception that no cocaine injections were given with the delivery of cue (tone/light).

#### Cue-Induced Reinstatement Test

Rats were returned to the same self-administration context and recorded the number of nosepokes to both operandi (active and inactive) for 1 h. The conditions were the same as during cocaine self-administration training except that cocaine injections were not given upon active nosepoking.

#### Priming-Induced Reinstatement Test

For priming-induced reinstatement test, rats were injected with cocaine (5 mg/kg, i. p.) 5 min before the start of the session. Rats then were placed in the same self-administration context and recorded the number of nosepokes to both operandi (active and inactive) was recorded for 1 h. The conditions were the same as during cocaine self-administration training except that active nosepokes were not reinforced with cocaine.

#### Spontaneous Recovery Test

For a spontaneous recovery test, active and inactive operandi nosepokes were recorded for 1 h after 28 days withdrawal. The testing conditions were similar to those during the cue-induced reinstatement test.

### Experimental Design

#### Experiment 1: Effect of Immediate Rapamycin Administration After Retrieval of Cocaine cue Memory on Subsequent cue-Induced + Priming-Induced Reinstatement

**FIGURE 1:**
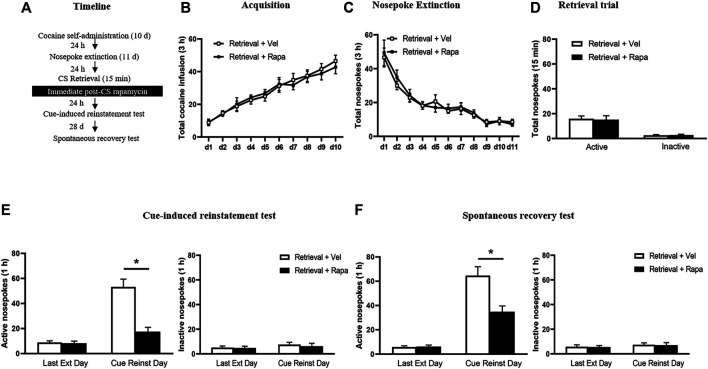
Rapamycin administration immediately after retrieval of cocaine cue memory reduces subsequent cue-induced reinstatement of cocaine seeking behavior. **(A)** Experimental procedure. **(B)** Total number of cocaine infusions across acquisition of cocaine self-administration sessions. **(C)** Total number of active nosepoke responses across nosepoke response extinction. **(D)** Nosepoke responses during retrieval trial. **(E)** Active **(left)** and inactive **(right)** nosepoke responses during the last session of the nosepoke extinction sessions and the cue-induced reinstatement test. **(F)** Active **(left)** and inactive **(right)** nosepoke responses during the last session of the cue extinction sessions and the priming-induced reinstatement test. *Different from the Retrieval + Veh group, *p <* 0.05.

Rats received three 1-h daily sessions of intravenous cocaine self-administration training for 10 days, and 24 h after training, all rats then underwent 11 consecutive days of daily nosepoke extinction training. 24 h after the last nosepoke extinction session, the rats were divided into two groups: 1) Intraperitoneal injection of rapamycin (10 mg/kg, i. p.) immediately after a 15 min retrieval trial (Retrieval + Rapa); 2) Intraperitoneal injection of vehicle (0.3 ml/kg, i. p.) immediately after a 15 min retrieval trial (Retrieval + Vel). The doses of rapamycin were chosen based on previous studies (Lin et al., 2014; Zubedat and Akirav, 2017). On Day 23, a cue-induced reinstatement test was performed to verify whether the administration of rapamycin immediately after retrieval of cocaine cue memory destroys the expression of cocaine cue memory in rats. 24 h after the cue-induced reinstatement test, rats received daily cue extinction session for two consecutive days. 24 h later, on Day 26, rats were tested for cocaine priming-induced reinstatement (Figure 1A).

#### Experiment 2: Effect of Immediate Rapamycin Administration After Retrieval of Cocaine cue Memory on Subsequent Cue-Induced Reinstatement and Spontaneous Recovery

**FIGURE 2:**
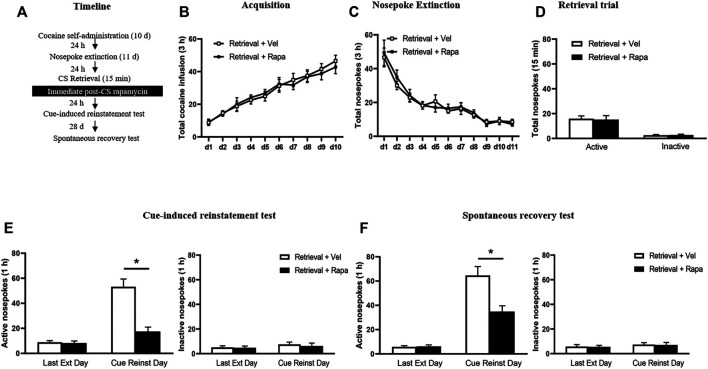
Rapamycin administration immediately after retrieval of cocaine cue memory decreases subsequent spontaneous recovery of cocaine seeking behavior. **(A)** Experimental procedure. **(B)** Total number of cocaine infusions across acquisition of cocaine self-administration sessions. **(C)** Total number of active nosepoke responses across nosepoke response extinction. **(D)** Nosepoke responses during retrieval trial. **(E)** Active **(left)** and inactive **(right)** nosepoke responses during the last session of the nosepoke extinction sessions and the cue-induced reinstatement test. **(F)** Active **(left)** and inactive **(right)** nosepoke responses during the last session of the cue extinction sessions and the spontaneous recovery test. *Different from the Retrieval + Veh group, *p <* 0.05.

Rats received three 1 h daily sessions of intravenous cocaine self-administration training for 10 days, and 24 h after training, all rats then underwent 11 consecutive days of daily nosepoke extinction training. 24 h after the last nosepoke extinction session, the rats were divided into two groups: 1) Intraperitoneal injection of rapamycin (10 mg/kg, i. p.) immediately after a 15 min retrieval trial (Retrieval + Rapa); 2) Intraperitoneal injection of vehicle (0.3 ml/kg, i. p.) immediately after a 15 min retrieval trial (Retrieval + Vel). 24 h after the last extinction session, rats underwent the cue-induced reinstatement test. To confirm the long-term inhibition effect of rapamycin on cocaine-seeking behavior in rats, 28 days after the cue-induced reinstatement test, all rats were tested for spontaneous recovery on Day 58 (Figure 2A).

#### Experiment 3: Effect of Rapamycin Treatment on Subsequent Cue-Induced + Priming-Induced Reinstatement in Non-retrieved Controls

**FIGURE 3:**
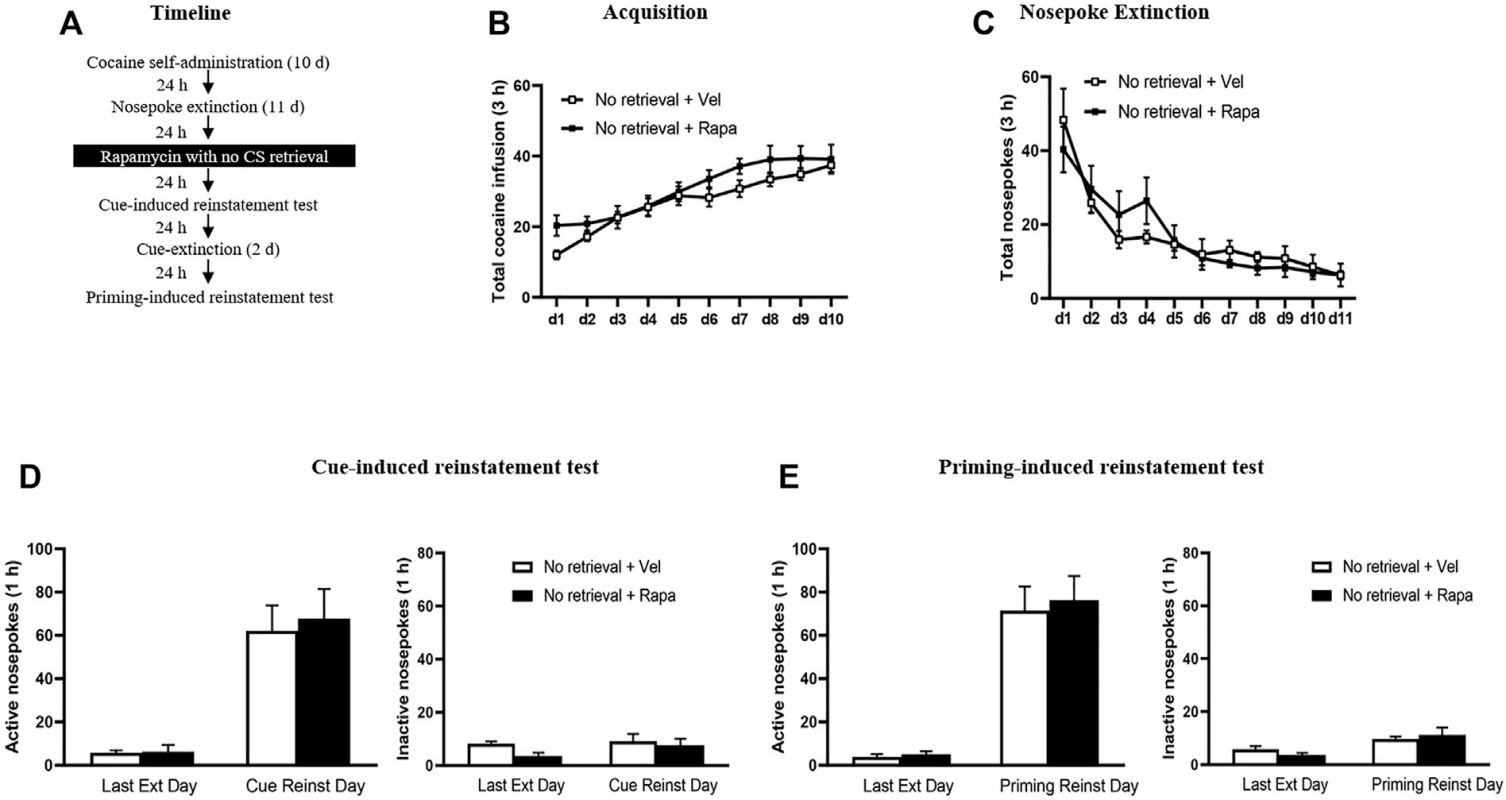
Rapamycin treatment without retrieval has no effects on subsequent reinstatement of cocaine seeking behavior. **(A)** Experimental procedure. **(B)** Total number of cocaine infusions across acquisition of cocaine self-administration sessions. **(C)** Total number of active nosepoke responses across nosepoke response extinction. **(D)** Active **(left)** and inactive **(right)** nosepoke responses during the last session of the nosepoke extinction sessions and the cue-induced reinstatement test. **(E)** Active **(left)** and inactive **(right)** nosepoke responses during the last session of the cue extinction sessions and the priming-induced reinstatement test.

The experimental procedure for Experiment 3 was the same as in Experiment 1, except that injection of rapamycin was made 24 h after the last nosepoke extinction session without the retrieval trial session (Figure 3A).

#### Experiment 4: Effect of Delayed Rapamycin Treatment After Retrieval of Cocaine Cue Memory on Subsequent Cue-Induced + Priming-Induced Reinstatement

**FIGURE 4:**
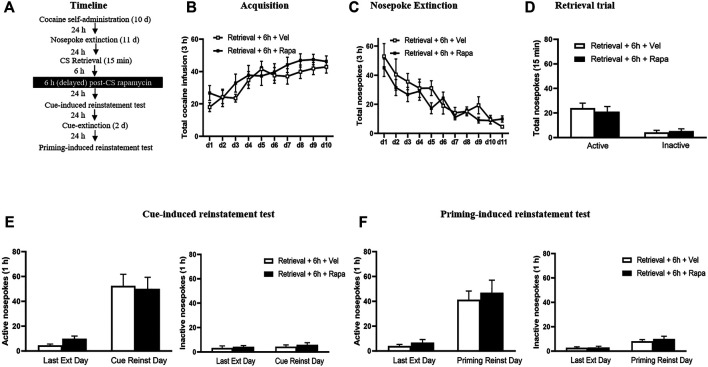
Rapamycin treatment 6 h after retrieval of cocaine cue memory has no effects on subsequent reinstatement of cocaine seeking behavior. **(A)** Experimental procedure. **(B)** Total number of cocaine infusions across acquisition of cocaine self-administration sessions. **(C)** Total number of active nosepoke responses across nosepoke response extinction. **(D)** Nosepoke responses during retrieval trial. **(E)** Active **(left)** and inactive **(right)** nosepoke responses during the last session of the nosepoke extinction sessions and the cue-induced reinstatement test. **(F)** Active **(left)** and inactive **(right)** nosepoke responses during the last session of the cue extinction sessions and the priming-induced reinstatement test.

The experimental procedure for Experiment 4 was the same as in Experiment 1, except that rapamycin was administered intraperitoneally 6 h after the retrieval trial session (Figure 4A).

### Statistical Analysis

The data were analyzed using repeated measures ANOVAs with between-subjects factor of treatment condition and within-subjects factor of test condition followed by Tukey’s *post-hoc* test in each experiment (see results). The values are presented as the mean ± S.E.M. Values of *p* < 0.05 were considered statistically significant.

## Results

### Experiment 1: Rapamycin Administration Immediately After Retrieval of Cocaine Cue Memory Reduces Subsequent Reinstatement of Cocaine-Seeking Behavior

In Experiment 1, we test the effect of immediate rapamycin administration after retrieval of cocaine cue memory on cue-induced and priming-induced reinstatement of cocaine seeking behaviors (Figure 1). Results were analyzed by repeated measures-ANOVA, with the treatment condition (Retrieval + Rapa, Retrieval + Veh) as a between-subjects factor and test condition as a within-subjects factor. There was no difference between two groups in the total numbers of cocaine infusions during acquisition (with the treatment condition as a between-subjects factor and the different training days as a within-subject factor) [main effect of the training day: F_(9,126)_ = 36.620, *p* < 0.001; main effect of the treatment condition: F_(1,14)_ = 0.035, *p* = 0.854; interaction of training day × treatment condition: F_(9_,_126)_ = 0.299, *p* = 0.974] (Figure 1B). Furthermore, statistical analysis of the number of active nosepokes in extinction session revealed no group difference in the Retrieval + Rapa group and the Retrieval + Veh group [main effect of the extinction day: F_(10,140)_ = 32.410, *p* < 0.001; main effect of the treatment condition: F_(1,14)_ = 0.508, *p* = 0.488; interaction of extinction day × treatment condition: F_(10,140)_ = 0.331, *p* = 0.971] (Figure 1C). For the retrieval trial, there were no group differences in the numbers of nosepokes [main effect of the different nosepokes: F_(1,14)_ = 42.61, *p* < 0.001; main effect of the treatment condition: F_(1,14)_ = 0.01549, *p* = 0.9027; interaction of different nosepokes × treatment condition: F_(1,14)_ = 0.03614, *p* = 0.8519] (Figure 1D). However, there was a significant difference in active nosepokes between two groups in the cue reinstatement tests [main effect of the test condition: F_(1,14)_ = 45.940, *p* < 0.001; main effect of the treatment condition: F_(1,14)_ = 28.342, *p* < 0.001; interaction of test condition × treatment condition: F_(1,14)_ = 19.710, *p* = 0.001]; *Post hoc* shown that drug-seeking in the Retrieval + Rapa group was significantly reduced compared to the Retrieval + Veh group in the cue-induced reinstatement test (*p* < 0.05) (Figure 1E left column). There was no significant difference in inactive nosepokes [main effect of the test condition: F_(1,14)_ = 1.575, *p* = 0.230; main effect of the treatment condition: F_(1,14)_ = 0.172, *p* = 0.684; interaction of test condition × treatment condition: F_(1,14)_ = 0.080, *p* = 0.781] (Figure 1E right column). For priming-induced reinstatement tests, repeated measures-ANOVA revealed a significant effect of active nosepokes [main effect of the test condition: F_(1,14)_ = 88.352, *p* < 0.001; main effect of the treatment condition: F_(1,14)_ = 11.98, *p* = 0.004; interaction of test condition × treatment condition: F_(1,14)_ = 10.560, *p* = 0.006; ]; *Post hoc* shown that drug-seeking in the Retrieval + Rapa group was significantly reduced compared to the Retrieval + Veh group in the priming-induced reinstatement test (*p* < 0.05) (Figure 1F left column), but no significant difference in inactive nosepokes [main effect of the test condition: F_(1,14)_ = 0.654, *p* = 0.432; main effect of the treatment condition: F_(1,14)_ = 0.066, *p* = 0.801; interaction of test condition × treatment condition: F_(1,14)_ = 0.001, *p* = 0.975] (Figure 1F right column). In summary, these results scxuggest that inhibition of mTOR kinase by rapamycin disrupts cocaine addiction memory and suppresses cue-induced and priming-induced reinstatement of cocaine-seeking behavior.

### Experiment 2: Rapamycin Administration Immediately After Retrieval of Cocaine Cue Memory Decreases Spontaneous Recovery of Cocaine-Seeking Behavior

In Experiment 2, we investigated the effect of rapamycin administration immediately after retrieval of cocaine cue memory on subsequent reinstatement and spontaneous recovery tests of cocaine seeking behaviors (Figure 2). The results were analyzed by repeated-measures ANOVA, with the treatment condition (Retrieval + Rapa, Retrieval Veh) as a between-subjects factor and the test condition as a within-subjects factor. There was no significant difference between the two groups in total numbers of cocaine infusions during acquisition (with the treatment condition as a between-subjects factor and the different training days as a within-subject factor) [main effect of the training day: F_(9,171)_ = 41.518, *p* < 0.001; main effect of the treatment condition: F_(1,19)_ = 0.077, *p* = 0.785; interaction of training day × treatment condition: F_(9,171)_ = 0.757, *p* = 0.656] (Figure 2B). In addition, statistical analysis of the numbers of active nosepokes during the extinction session revealed no significant group differences between the Retrieval + Rapa group and the Retrieval + Veh group [main effect of the extinction day: F_(10,190)_ = 30.187, *p* < 0.001; main effect of the treatment condition: F_(1,19)_ = 0.193, *p* = 0.665; interaction of extinction day × treatment condition: F_(10,190)_ = 0.265, *p* = 0.988] (Figure 2C). For the retrieval trial, there were no group differences in the numbers of nosepokes [main effect of the different nosepokes: F_(1,19)_ = 29.68, *p* < 0.001; main effect of the treatment condition: F_(1,19)_ = 0.2486, *p* = 0.6238; interaction of different nosepokes × treatment condition: F_(1__19)_ = 0.0484, *p* = 0.8282] (Figure 2D). However, there was a significant difference in active nosepokes between these groups in the cue-induced reinstatement test [main effect of the test condition: F_(1, 18)_ = 55.265, *p* < 0.001; main effect of the treatment condition: F_(1,18)_ = 19.091, *p* < 0.001; interaction of test condition × treatment condition: F_(1,18)_ = 14.283, *p* = 0.001]; *Post hoc* shown that drug-seeking in the Retrieval + Rapa group was significantly reduced as compared to the Retrieval + Veh group during the cue-induced reinstatement test (*p* < 0.05) (Figure 2E left column), but no significant difference in inactive nosepokes [main effect of the test condition: F_(1,19)_ < 0.001, *p* = 0.989; main effect of the treatment condition: F_(1,19)_ = 0.295, *p* = 0.593; interaction of test condition × treatment condition: F_(1,19)_ = 0.085, *p* = 0.773] (Figure 2E right column). Furthermore, repeated-measures ANOVA revealed a significant effect of active nosepokes in the spontaneous recovery test [main effect of the test condition: F_(1,19)_ = 60.320, *p* < 0.001; main effect of the treatment condition: F_(1,19)_ = 11.220, *p* = 0.003; interaction of test condition × treatment condition: F_(1,19)_ = 15.444, *p* = 0.001]; *Post hoc* shown that drug-seeking in the Retrieval + Rapa group was significantly reduced as compared to the Retrieval + Veh group in the spontaneous recovery test (*p* < 0.05) (Figure 2F left column), but no significant difference in inactive nosepokes [main effect of the test condition: F_(1,19)_ = 0.987, *p* < 0.001; main effect of the treatment condition: F_(1,19)_ = 0.150, *p* = 0.703; interaction of test condition × treatment condition: F_(1,19)_ = 0.012, *p* = 0.915] (Figure 2F right column). These results indicate that inhibition of mTOR kinase by rapamycin treatment disrupts cocaine addiction memory and suppresses spontaneous recovery of cocaine-seeking behavior.

### Experiment 3: Rapamycin Treatment Without Retrieval has No Effects on Subsequent Reinstatement of Cocaine-Seeking Behavior

In Experiment 3, we examined whether rapamycin disrupting reconsolidation of cocaine memory was retrieval dependent. After the cocaine self-administer acquisition and extinction, rats administered rapamycin or vehicle but without a retrieval trial (Figure 3). There was no significant difference between the groups in the total numbers of cocaine infusions during acquisition (with the treatment condition as a between-subjects factor and the different training days as a within-subject factor) [main effect of the training day: F_(9,144)_ = 29.498, *p* < 0.001; main effect of the treatment condition: F_(1,16)_ = 2.014, *p* = 0.175; interaction of training day × treatment condition: F_(9,144)_ = 0.943, *p* = 0.490] (Figure 3B). Furthermore, statistical analysis on the number of active nosepokes during the extinction session revealed that there were no group difference in the Retrieval + Rapa group and the Retrieval + Veh group [main effect of the extinction day: F_(10,160)_ = 18.906, *p* < 0.001; main effect of the treatment condition: F_(1,16)_ = 0.044, *p* = 0.837; interaction of extinction day × treatment condition: F_(10,160)_ = 0.735, *p* = 0.691] (Figure 3C). In addition, there was no significant difference in active nosepokes between these groups during the reinstatement tests [main effect of the test condition: F_(1, 16)_ = 39.240, *p* < 0.001; main effect of the treatment condition: F_(1,16)_ = 0.122, *p* = 0.732; interaction of test condition × treatment condition: F_(1,16)_ = 0.074, *p* = 0.789] (Figure 3D left column), no significant difference in inactive nosepokes [main effect of the test condition: F_(1,16)_ = 1.493, *p* = 0.239; main effect of the treatment condition: F_(1,16)_ = 2.419, *p* = 0.139; interaction of test condition × treatment condition: F_(1,16)_ = 0.620, *p* = 0.443] (Figure 3D right column). For priming-induced reinstatement tests, repeated-measures ANOVA revealed no significant effect of active nosepokes [main effect of the test condition: F_(1,16)_ = 75.577, *p* < 0.001; main effect of the treatment condition: F_(1,16)_ = 0.152, *p* = 0.701; interaction of test condition × treatment condition: F_(1,16)_ = 0.053, *p* = 0.821] (Figure 3F left column), no significant difference in inactive nosepokes [main effect of the test condition: F_(1,16)_ = 10.826, *p* = 0.005; main effect of the treatment condition: F_(1,16)_ = 0.031, *p* = 0.862; interaction of test condition × treatment condition: F_(1,16)_ = 1.111, *p* = 0.307] (Figure 3F right column). There is no significant difference between the groups in all tests (all *p* > 0.05). Therefore, the results of Experiment 3 indicate that the effect of rapamycin in Experiment 1 depends on the retrieval trial and that rapamycin administration without a retrieval trial has no effects on subsequent cue-induced and priming-induced reinstatement of cocaine-seeking behavior.

### Experiment 4: Delayed Rapamycin Treatment Following Retrieval of Cocaine Cue Memory has No Effects on Subsequent Reinstatement of Cocaine-Seeking Behavior

In Experiment 4, we examined whether the rapamycin treatment outside the sensitive time window could disrupted the reconsolidation of cocaine memory. We assessed the effect of rapamycin administration 6 h after retrieval of cocaine cue memory on cue-induced and priming-induced reinstatement of cocaine seeking behaviors (Figure 4). During the acquisition sessions, there was no significant difference between two groups in the total numbers of cocaine infusions (with the treatment condition as a between-subjects factor and the different training days as a within-subject factor) [main effect of the training day: F_(9,117)_ = 15.687, *p* < 0.001; main effect of the treatment condition: F_(1,13)_ = 0.701, *p* = 0.418; interaction of training day × treatment condition: F_(9,117)_ = 1.021, *p* = 0.427] (Figure 4B). Furthermore, statistical analysis of the numbers of active nosepokes during the extinction sessions revealed no group difference in the Retrieval + Rapa group and the Retrieval + Veh group [main effect of the extinction day: F_(10,130)_ = 17.667, *p* < 0.001; main effect of the treatment condition: F_(1,13)_ = 1.088, *p* = 0.316; interaction of extinction day × treatment condition: F_(10,130)_ = 1.080, *p* = 0.383] (Figure 4C). For the retrieval trial, there were no group differences in the numbers of nosepokes [main effect of the different nosepokes: F_(1,13)_ = 57.10, *p* < 0.001; main effect of the treatment condition: F_(1,13)_ = 0.06455, *p* = 0.8034; interaction of different nosepokes × treatment condition: F_(1_,_13)_ = 0.7395, *p* = 0.4054] (Figure 4D). For the reinstatement tests, there was no differences in both active [main effect of the test condition: F_(1,13)_ = 49.409, *p* < 0.001; main effect of the treatment condition: F_(1,13)_ = 0.040, *p* = 0.845; interaction of test condition × treatment condition: F_(1,13)_ = 0.382, *p* = 0.547] (Figure 4E left column), and inactive [main effect of the test condition: F_(1,13)_ = 0.693, *p* = 0.420; main effect of the treatment condition: F_(1,13)_ = 0.620, *p* = 0.445; interaction of test condition × treatment condition: F_(1,13)_ = 0.028, *p* = 0.870] (Figure 4E right column) nosepokes between the two groups. For priming-induced reinstatement tests, repeated-measures ANOVA revealed no significant effect of active nosepokes [main effect of the test condition: F_(1,13)_ = 43.008, *p* < 0.001; main effect of the treatment condition: F_(1,13)_ = 0.349, *p* = 0.565; interaction of test condition × treatment condition: F_(1,13)_ = 0.058, *p* = 0.814] (Figure 4F left column), and no significant difference in inactive nosepokes [main effect of the test condition: F_(1,13)_ = 11.956, *p* = 0.004; main effect of the treatment condition: F_(1,13)_ = 0.690, *p* = 0.421; interaction of test condition × treatment condition: F_(1,13)_ = 0.204, *p* = 0.659] (Figure 4F right column). There is no significant difference between the groups in all tests (all *p* > 0.05). These findings indicate that the effect of mTOR kinase inhibition via rapamycin on blocking reinstatement of cocaine seeking is time-limited, that is, this effect must occur within 6 h after retrieval.

## Discussion

The study first identified the critical role of mTOR in reconsolidation in the classic cocaine cue memory model. The main findings of this study are following: 1) Systemic administration of rapamycin immediately after retrieval of cocaine cue memory effectively reduced the cocaine-seeking behavior induced by drug-associated cues and the reinstatement of cocaine seeking induced by cocaine in rats; 2) The inhibitory effect of systemic rapamycin administration immediately after retrieval of cocaine cue memory on cocaine-seeking behavior persisted for least 28 days; 3) rapamycin when administered intraperitoneally with a 6 h delay or without a retrieval trial had no effects on cue-induced and cocaine-priming-induced reinstatement of cocaine-seeking behavior, indicating that the inhibitory effects of rapamycin on cocaine-seeking behavior is retrieval-dependent and temporal-specific.

In the current study, we utilized a classic intravenous cocaine self-administration paradigm to investigate the effect of rapamycin on drug memory reconsolidation. After establishing a stable pattern of cocaine self-administration, nosepoke behavior was extinguished. According to previous studies, nosepoke behavior in two operandi did not accompanied by light/tone stimulus during nosepoke extinction sessions, while during the retrieval trial session, the rats were exposed to the training chamber for 15 min and active nosepoke behavior was accompanied by tone/light stimulus (Lee et al., 2005; Sanchez et al., 2010; Wells et al., 2013; Shi et al., 2015). We have demonstrated that intraperitoneal injection of rapamycin immediately after memory retrieval inhibits mTOR activity. On the following test day, we found that inhibiting mTOR activity reduces the reinstatement of cocaine-seeking behavior induced by cued, subsequent cocaine-priming, and spontaneous recovery. However, inhibition of mTOR activity did not affect subsequent cue-induced behavior and cocaine-priming-induced cocaine-seeking when administered with a 6 h delay or without retrieval. These results indicate that the administration of rapamycin immediately after retrieval of cocaine cue memory disrupts the reconsolidation process of cocaine cue memory, rather than it inhibits the expression of cocaine cue memory. Growing evidence demonstrates that reconsolidation is a *de novo* protein-dependent process, which is completed within 6 h after retrieval. The effectiveness of interventions that destroy reconsolidation depends on the time window and the manipulation of memory retrieval (Nader et al., 2000; Alberini, 2005; Dudai, 2006; Luo et al., 2015). Our results indicate that inhibition of mTOR activity by rapamycin on the destruction of cocaine cue memory is retrieval-dependent and temporal-specific. These findings are consistent with the memory reconsolidation theory. These behavioral experiments demonstrate that the activity of mTOR mediates the reconsolidation of cocaine cue memory, and such process can be disrupted by rapamycin leading to a reduction of cocaine-seeking and prevention of relapse. However, the limitation of the current study is that we use systemic injections of rapamycin to block reconsolidation, which can’t identify the mTOR activity of specific brain areas is involved in the reconsolidation of cocaine cue memory using the self-administration rats model. Previous studies have been shown that basolateral amydala (BLA) and nucleus accumbens core (NAcc) is critically involved in the reconsolidation of drug memories (Lee et al., 2005; Sanchez et al., 2010; Otis et al., 2015; Shi et al., 2015; Rich et al., 2016; Hafenbreidel et al., 2017; Bender B. N. and Torregrossa M. M., 2020). Considering mTOR is extensively expressed in the above mentioned brain regions (Caron et al., 2015) and rapamycin could cross the brain-blood barrier (Cloughesy et al., 2008), systemic injection of rapamycin could inhibit the activity of mTOR in the BLA and NAc. Therefore, the activity of mTOR in amygdala and/or NAc could be required for the reconsolidation of cocaine cue memories (Luo et al., 2013; Bender B. N. and Torregrossa M. M., 2020). Our future study will use microinjection method to identify the mTOR activity of specific brain areas is required for reconsolidation of cocaine cue memory in the rat self-administration model.

The mTOR signaling pathway regulates the downstream eukaryotic translation initiation factor, the eIF4E-binding protein 1 (4E-BP1) and ribosomal protein S6 kinases (S6Ks), it thus plays a critical role in the protein translation process (Peterson et al., 1999; Choi et al., 2002). mTOR alters the binding state of eIF4E and 4E-BP1 phosphorylation level to regulate the initiation of eukaryotic protein translation (Proud, 2007). mTOR activation increases the phosphorylation level of downstream S6Ks and promotes peptide chain elongation during protein translation (Wang et al., 2001; Holz et al., 2005). Hence, the mTOR signaling pathway regulates protein synthesis in eukaryotic cells via regulating the activity of downstream target proteins 4E-BP1 and S6Ks. Reconsolidation is a protein synthesis-dependent process, thus the mTOR signaling pathway may regulate reconsolidation of cocaine addiction memory through these mechanisms.

In addition, the mTOR signaling pathway can also affect the protein’s translation, synthesis, apoptosis, and the occurrence and proliferation of tumor cells by regulating ribosome synthesis and downstream gene transcription (Sabatini et al., 1994). Furthermore, the activity of mTOR can also regulate downstream protein kinases, such as the activity of Akt and protein kinase C (PKC), and regulate the biosynthesis and metabolism of cytoskeleton-related proteins (Sarbassov et al., 2004; Sarbassov et al., 2005). Meanwhile, the protein kinases including PKA, PKC, Akt, and biosynthesis and metabolism of cytoskeleton protein have been found to be involved in reconsolidation (Milton and Everitt, 2010; Neasta et al., 2010; Sanchez et al., 2010; Neasta et al., 2014; Ben Hamida et al., 2019; Morisot et al., 2019; Bender B. N. and Torregrossa M. M., 2020). Thus, we speculate that the mTOR signaling pathway may regulate the reconsolidation of cocaine addiction memory by affecting the protein kinase system and cytoskeleton proteins. Consistently, some previous studies have demonstrated that changes in synaptic plasticity mediated by mTORC1 and its downstream target proteins regulate alcohol intake and drug-seeking behavior in rats (Neasta et al., 2010; Neasta et al., 2014; Ben Hamida et al., 2019; Morisot et al., 2019).

Previous studies have also shown that immediate intraperitoneal rapamycin injection after reactivating memory by exposure to drug-associated environment can disrupt reconsolidation of cocaine, morphine, and alcohol-induced addiction memory in the CPP conditioned place preference paradigm (Lin et al., 2014). In the alcohol self-administration paradigm, it was also found that the activity of mTORC1 in CeA was increased after memory reactivation, and inhibition of mTOR1 activity in CeA with rapamycin was reported to destroy reconsolidation of alcohol addiction memory causing a reduction in reinstatement of alcohol-seeking behavior in rats (Barak et al., 2013). Both CPP and SA paradigms are classic animal models used to investigate addiction memory, but there are essential differences between them. The CPP model is based on classical conditioning learning and memory model, while the self-administration is based on operant conditioning. During the CPP conditioning, the experimental animals passively receive addictive drugs, and the dose is relatively lower as compared to the SA model. In intravenous self-administration model, experimental animals need to actively complete certain operational behaviors to obtain addictive drugs (de Wit and Stewart, 1981; Cami and Farre, 2003). Therefore, the SA paradigm mimics the characteristics of human drug addiction more closely that the CPP paradigm. In addition, a previous study found that microinjections of rapamycin into the nucleus accumbens (NAc) 30 min before the cocaine cue-induced reinstatement test inhibits the expression of drug-seeking behavior induced by the drug-associated cue in SA model. In this study, rapamycin was administered before the cue-induced drug-seeking behavior test, which verified the role of mTOR activity in the expression of cocaine addiction memory in rats. It has also been shown that exposure to the cocaine-associated environment increases the mTOR activity and the phosphorylation level of the p70s6k-rps6 downstream pathway in the rat NAc core, but not the NAc shell (Wang et al., 2010). Our results indicate that reactivation of cocaine addiction memory activates the mTOR signaling pathway, and we can deduce that the mTOR signaling pathway is involved in memory reconsolidation. Consistent with these previous studies, the present research further identifies the critical role of the mTOR signaling pathway in reconsolidation of addiction memory using the operational cocaine cue memory model.

In summary, our findings demonstrated that the mTOR activity plays a critical role in the reconsolidation of cocaine cue memory using the operational cocaine SA paradigm. Administration of rapamycin immediately after memory reactivation disrupts reconsolidation of the cocaine cue memory, leading to a reduction in reinstatement of cocaine seeking behavior in rats. The present study identifies the mTOR signaling pathway as a promising target for treating cocaine addiction and preventing relapse.

## Data Availability

The raw data supporting the conclusions of this article will be made available by the authors, without undue reservation.
